# Advances and Insights of APC-Asef Inhibitors for Metastatic Colorectal Cancer Therapy

**DOI:** 10.3389/fmolb.2021.662579

**Published:** 2021-04-22

**Authors:** Xiuyan Yang, Jie Zhong, Qiufen Zhang, Li Feng, Zhen Zheng, Jian Zhang, Shaoyong Lu

**Affiliations:** ^1^Department of Pathophysiology, Key Laboratory of Cell Differentiation and Apoptosis of Chinese Ministry of Education, Shanghai Jiao Tong University School of Medicine, Shanghai, China; ^2^Medicinal Chemistry and Bioinformatics Center, Shanghai Jiao Tong University School of Medicine, Shanghai, China

**Keywords:** APC-Asef inhibitors, structure based drug design, peptide inhibitors, small molecule inhibitors, colorectal cancer therapy

## Abstract

In Colorectal cancer (CRC), adenomatous polyposis coli (APC) directly interacts with the Rho guanine nucleotide exchange factor 4 (Asef) and releases its GEF activity. Activated Asef promotes the aberrant migration and invasion of CRC cell through a CDC42-mediated pathway. Knockdown of either APC or Asef significantly decreases the migration of CRC cells. Therefore, disrupting the APC-Asef interaction is a promising strategy for the treatment of invasive CRC. With the growth of structural information, APC-Asef inhibitors have been designed, providing hope for CRC therapy. Here, we will review the APC-Asef interaction in cancer biology, the structural complex of APC-Asef, two generations of peptide inhibitors of APC-Asef, and small molecule inhibitors of APC-Asef, focusing on research articles over the past 30 years. We posit that these advances in the discovery of APC-Asef inhibitors establish the protein-protein interaction (PPI) as targetable and provide a framework for other PPI programs.

## Introduction

Colorectal cancer (CRC), which results from the complex factors of genes and the environment, is the world’s fourth most deadly cancer. The incidence of CRC is increasing with the rapid economic growth and global aging population (Steffen, 1986; Lise et al., 1987; Kemeny, 1992; Weekes et al., 2009). In the early stage of CRC, the 5-year survival rate is up to 90 percent after surgical excision. In contrast, those with the advanced stages usually need to treat with combination therapy of chemotherapy and surgery, and the 5-year survival rate presents a decline to varying degrees. Despite the dramatic improvement in CRC treatment, metastatic CRC is still an unmet challenge (Gallina et al., 2006; Aliev and Aliev, 2007; Haggar and Boushey, 2009; Zheng et al., 2014; Liu et al., 2015; Jideh and Bourke, 2018). Therefore, there is an urgent need for innovative and effective drug candidates, especially for metastatic CRC patients.

**FIGURE 1:**
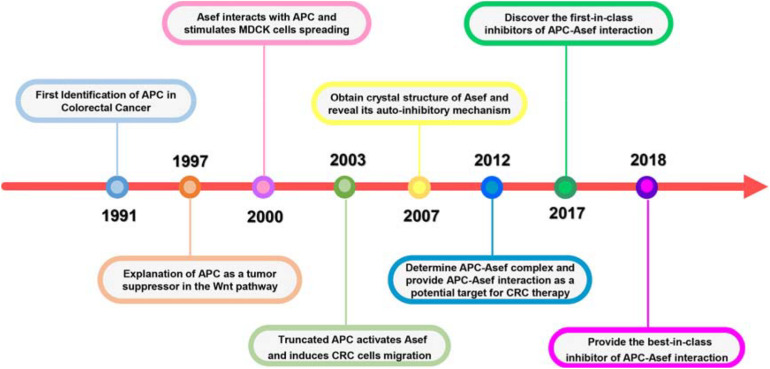
Significant milestones in the history of APC-Asef Interaction.

The Adenomatous polyposis coli (APC) protein plays a pivotal role in sporadic and familial CRCs. APC was first characterized as a mutated gene in CRC by Groden et al. (1991; Figure 1). The following work explained how APC exerted its effect as a tumor suppressor in the traditional Wnt signaling pathway. APC could form APC/Axin/GSK3-β destruction complex and downregulate β-catenin. Mutated APC resulted in the aberrant stabilization and accumulation of β-catenin, which could activate the transcription of the TCF/LEF family transcription factors associated with proliferation (Korinek et al., 1997; Morin et al., 1997; He et al., 1998; Tetsu and McCormick, 1999). APC contains multiple domains and interacts with other proteins, suggesting APC act in non-traditional roles outside the Wnt pathway in CRC. Kawasaki et al. identified Rho guanine nucleotide exchange factor 4 (Asef) released its auto-inhibited activity by interacting with the armadillo repeat domain (ARM) of APC, thereby stimulating Asef-mediated MDCK cell flattening, lamellipodia formation, and cell spreading (Kawasaki et al., 2000). In addition, the expression of truncated APC could activate Asef in CRC, inducing inappropriate cell migration and invasion (Kawasaki et al., 2003). In 2007, the crystal structure of Asef was solved and demonstrated its auto-inhibitory mechanism caused by the intramolecular interaction between the SH3 domain and DH domain (Murayama et al., 2007). Zhang et al. (2012) reported the APC-Asef complex’s structures and elucidated the molecular mechanism of Asef recognition by APC, which provided APC-Asef interaction as a potential target for CRC therapy (Zhang et al., 2012). Since the characterization of the APC-Asef co-complex, remarkable progress has been made in the development of APC-Asef inhibitors. Based on these structures, MAIT-203, the first-in-class inhibitor of APC-Asef interaction, was generated, which could efficiently reduce the migration and invasion of CRC cells by blocking APC-Asef interaction (Jiang et al., 2017). After multiple optimizations, MAI-400, the best-in-class inhibitor of APC-Asef interaction, was discovered with an IC_50_ of 250 nM (Yang et al., 2018). This article reviews these processes and breakthroughs on APC-Asef interaction as a valuable target for CRC therapy. We hope that it will conduct a longitudinal analysis of critical issues, limitations, and opportunities to develop new CRC candidates and make significant contributions to medical research.

## APC-Asef Interaction Promotes Colorectal Cancer Cell Migration and Cancer Progression

**FIGURE 2:**
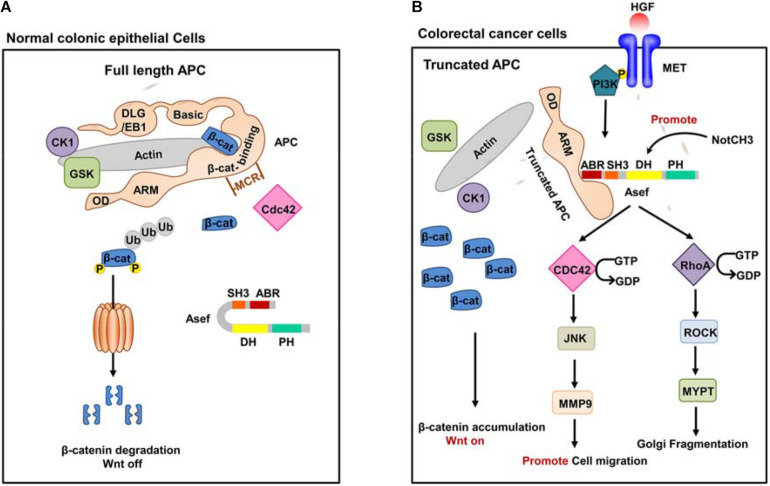
The function of Wile-type APC and truncated APC. **(A)** Wild-type full-length APC in normal colonic epithelial cells. In normal colonic epithelial cells, APC acts as a tumor suppressor in the traditional Wnt signaling pathway. APC could interact with β-catenin and form APC/Axin/GSK3-β destruction complex. The destruction complex promotes phosphorylation of β-catenin and induces its proteasome-mediated degradation, thus suppressing the canonical Wnt signaling pathway. **(B)** A representative truncated APC in CRC cells. In CRC cells, truncated APC interacts with Asef instead of β-catenin and stimulates the GEF activity of Asef. Activated Asef promotes cell migration and Golgi fragmentation through the downstream pathway.

APC is a multifunctional protein, which plays crucial and diverse functions in cellular processes, including Wnt/β-catenin signaling (Ha et al., 2004; Xing et al., 2004; MacDonald et al., 2009; Wang et al., 2014) and non-traditional Wnt pathway (Hamada and Bienz, 2002; Bienz and Hamada, 2004; Akiyama and Kawasaki, 2006; Etienne-Manneville, 2009; Figure 2). The human APC protein mainly contains an oligomerization domain (OD), an ARM domain, a β-catenin binding region, a basic domain, and an end-binding protein 1 (EB1)/ discs large (DLG) binding domain (Figure 2A). APC can interact with various proteins, including β-catenin, Asef, IQGAP1, Sam68, KAP-3, Kinesin-2, and Amer1. APC binds to β-catenin and recruits it to a destruction complex that contains axin, glycogen synthase kinase 3β (GSK3β), and casein kinase 1α. The destruction complex promotes the phosphorylation of β-catenin and induces its proteasome-mediated degradation, thus suppressing the canonical Wnt signaling pathway (Figure 2A; Li et al., 1998; Sieber et al., 2000; Zhang et al., 2011). Other PPIs are investigated to elucidate the function of APC in non-traditional pathways comprehensively. APC’s interaction with Asef stimulates the GEF activity of Asef and promotes Asef-mediated cell flatting, cell membrane ruffling, lamellipodia formation, and cell spreading (Kawasaki et al., 2000). APC also interacts with IQGAP1 at the leading edge of migrating cells to regulate cell polarization and migration (Watanabe et al., 2004). APC can bind to the tyrosine-rich domain of Sam68 and regulate alternative splicing of TCF-1 (Morishita et al., 2011). APC interacts with KAP-3 to form a complex with β-catenin and the kinesin superfamily proteins KIF3A-KIF3B, leading to increased cell migration (Mahajan et al., 2015). The interaction between APC and Kinesin-2 plays an essential role at dendrite branch points to resolve microtubule collisions (Weiner et al., 2016). APC interacts with Amer1 and regulates the APC-dependent maintenance of intercellular junctions and negatively regulates the Wnt/β-catenin signal transduction pathway (Grohmann et al., 2007; Tanneberger et al., 2011; Zhang et al., 2015). These PPIs explain the function of APC in tumor formation and migration.

APC mutations have been identified in most sporadic and family CRCs and are considered an early event for tumorigenesis. The presence of APC mutations in premalignant lesions detected by exome sequencing of human colorectal adenomas and the surrounding mucosa confirmed its role in the early growth of colon adenoma. APC mutant mouse models have proved that the alterations in the APC gene are essential for the initiation of intestinal tumor (Miyoshi et al., 1992; Sen-Gupta et al., 1993; Su et al., 1993; Iwama et al., 1999; Iwama, 2001; Nikolaev et al., 2012; Borras et al., 2016; Zhang and Shay, 2017). Most APC mutations are confined to their central region, referred to as the mutation cluster region (MCR). Alterations in the APC gene generate C-terminal truncated APC fragment and cause loss of the domains required for binding to β-catenin and microtubules. Therefore, truncated APC is disabled to degrade β-catenin, resulting in the accumulating of β-catenin, which constitutively activates the Wnt signaling pathway in CRC cells (Tominaga et al., 1998; Norris et al., 2000; Schneikert and Behrens, 2006; Schneikert et al., 2007, 2011). In addition, the truncated APC effectively interacts with Asef via its ARM domain (Figure 2B). Asef, one of the human Dbl-family of guanine nucleotide exchange factors (GEFs), consists of an APC-binding region (ABR), an Src homology 3 (SH3) region, a Dbl homology (DH) region, and a pleckstrin homology (PH) region. Asef is usually auto-inhibited by binding between its SH3 domain and DH-PH domains (Kawasaki et al., 2000; Gotthardt and Ahmadian, 2007; Mitin et al., 2007; Murayama et al., 2007; Muroya et al., 2007; Yoshihiro et al., 2008). Zhang et al. (2012) suggested that the intramolecular interaction strength between the DH and SH3 domain of Asef was less than that of Asef-APC. The isothermal titration calorimetry (ITC) assay revealed a tight binding between APC and Asef with a *K*_*d*_ value of 17.8 nM. Therefore, the interaction of the Asef-DH and Asef-SH3 was easily separated once APC binding with Asef.

Upon binding to the truncated APC, Asef releases its auto-inhibition and stimulates its GEF activity, thereby catalyzing the exchange of GDP for GTP in CDC42. The activated Asef and CDC42 upregulate the expression of matrix metalloproteinase 9 (MMP9) mainly through the c-Jun N-terminal kinase (JNK) pathway, thereby stimulating cell flattening, membrane ruffing, and lamellipodia formation and ultimately promoting cancer cell migration (Kawasaki et al., 2000, 2003, 2009a; Gotthardt and Ahmadian, 2007; Mitin et al., 2007; Murayama et al., 2007; Itoh et al., 2008).

It has also been shown that epidermal growth factor (EGF), hepatocyte growth factor (HGF), and basic fibroblast growth factor (bFGF) increase the amount of the APC-Asef complex and induce its accumulation and co-localization in membrane ruffles and lamellipodia, thereby promoting cell migration (Itoh et al., 2008; Kawasaki et al., 2009a,b). In addition, upstream PI3K may affect APC and Asef in the HGF signaling pathway (Kawasaki et al., 2009b). These increase the possibility that HGF and PI3K activate the migration activity of CRC cells by inducing the accumulation of APC-Asef complex in membrane ruffles and lamellipodia. Additionally, the miR-1-NOTCH3 signaling plays a critical role in CRC cell migration and cancer angiogenesis by up-regulating Asef expression (Furukawa et al., 2013).

Recently, Kim et al. found that truncated APC activated Asef and then induced Golgi fragmentation by stimulating the RhoA-ROCK-MLC2 pathway. Golgi fragmentation led to the loss of cell polarity and disordered direction of cell migration, which facilitated CRC initiation and progression. This phenomenon may be an early event in CRC development, which affects the regular directed cell migration, promoting cancer initiation and progression (Rodriguez-Cruz et al., 2018).

In conclusion, truncated APC is disabled to play its suppressor role but constitutively activates Asef, promoting the migration of CRC cells and the progression to invasive malignancy.

## Disrupting APC-Asef Interaction Could Be a Potent Target for CRC Therapy

Recently, PPIs are becoming ponderable therapeutic targets for various diseases. APC-Asef interaction plays an essential role in the progression, invasion, and metastasis of CRC. Whether knocking down APC or Asef, it significantly decreased the migration of several types of CRC cells, such as HCT116 and SW480 (Kawasaki et al., 2003). Further study showed that the number and size of the intestinal adenomas in Apc^Min/+^ mice were significantly reduced (Kawasaki et al., 2009a). These results suggest that the APC-Asef interaction has an essential impact on intestinal adenoma formation and tumor progression. Therefore, the APC-Asef interaction might be a novel target for the treatment of CRC. APC-Asef inhibitors might provide therapeutic options for the treatment of metastatic CRC.

## Structure Characterization of APC-Asef Interaction

**FIGURE 3:**
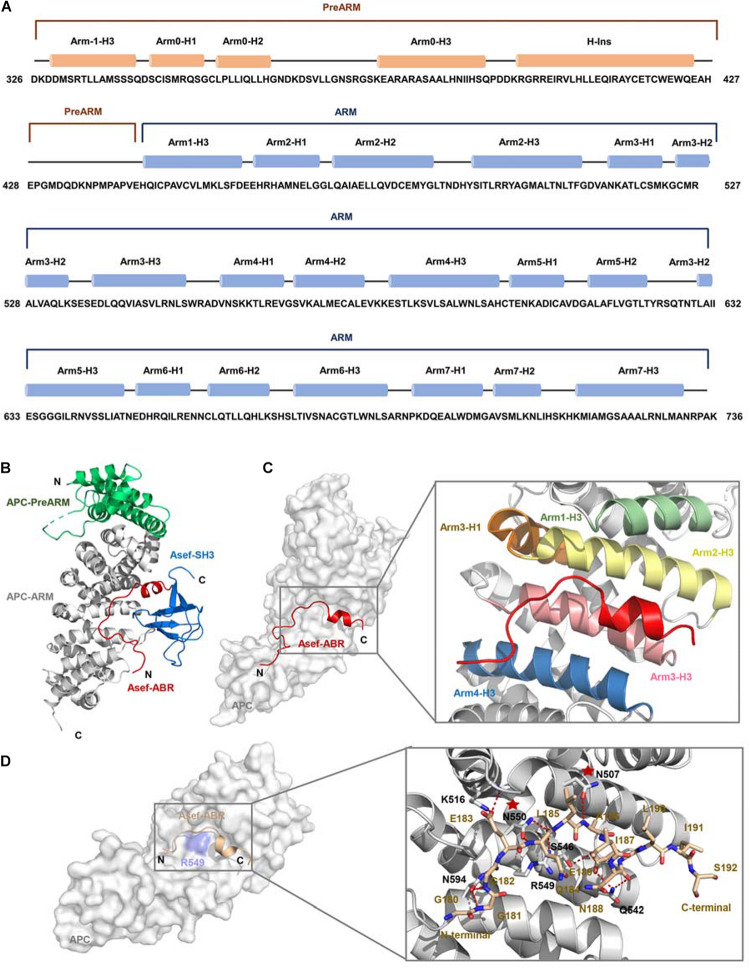
The crystal structure of APC and Asef **(A)** Sequence of human APC (residues 326-736). APC-PreARM residues (326-442) are labeled orange, and APC-ARM (453-767) are labeled blue. APC-PreARM consists of five α helices: Arm-1-H3, Arm0-H1, Arm0-H2, Arm0-H3, and the long helix insertion (H-Ins). APC-ARM contains seven armadillo repeats Arm1 to Arm7. **(B)** APC (PreARM-ARM)/Asef (ABR-SH3) complex structure, the PreARM portion of APC is shown in green, and the ARM portions of APC are shown in gray. The ABR domain of Asef is shown in red, and the SH3 domain of Asef is shown in blue. **(C)** Detailed information of the interface of APC and Asef complex. Asef is shown in red. Arm1-H3, Arm2-H3, Arm3-H1, Arm3-H3, and Arm4-H3 are shown in green, yellow, orange, pink, and blue, respectively. **(D)** Left: The interactions of APC and Asef-ABR. The ABR domain of Asef is shown in champagne, and APC is shown in gray. Right: Close-up view of interactions between APC and Asef. APC is shown as a gray carton, and Asef is shown as a champagne stick. Red dashed lines represent hydrogen bonds. The red stars represent APC-Asn507 and APC-Asn550 are critical in the interaction of APC and Asef.

Based on the critical influence of APC-Asef on the CRC migration, Zhang et al. (2015) attempted to solve the crystal structure of APC-Asef to provide a structural basis for drug development. The ARM domain of APC (residues 443-767), consisting of seven armadillo repeats, interacts with the ABR region of Asef. Another conserved region preceding the seven armadillo repeats was referred to as the PreARM region (residues 326-442). In addition, APC mutations usually result in a truncated APC fragment with intact PreARM and ARM in CRCs. The entire APC-PreARM-ARM region might form an ARM. Therefore, they determined the crystal structure of APC-PreARM-ARM (PDB: 3NMW), APC-PreARM-ARM/Asef-ABR-SH3 (PDB: 3NMX), and APC-PreARM-ARM/Asef-ABR (PDB: 3NMZ) (Zhang et al., 2012). Zhang et al. (2015) first detailed the APC-PreARM domain (residues 326-442), which consisted of four α helices (Arm-1-H3, Arm0-H1, Arm0-H2, and Arm0-H3) and a long helix insertion (H-Ins) (Figure 3A). The APC-PreARM domain packed widely with the ARM domain of APC, and together formed a single-folding unit (Figure 3B). The APC-PreARM-ARM/Asef-ABR-SH3 complex structure revealed that Asef-ABR bound to the concave surface of the APC-ARM, formed by the Arm1-H3, Arm2-H3, Arm3-H3, Arm4-H3, and Arm3-H1 (Figure 3C). However, the resolution is only 3.0 Å, so the APC-PreARM-ARM/Asef-ABR complex with a higher resolution (2.3 Å) was determined (Figure 3D) to obtain the precise binding information. The binding interface between APC and Asef is up to 1,300 Å. Many hydrogen bond networks and van der Waal contacts in APC-ARM/Asef-ABR complex interface stabilize the interaction of APC and Asef. The mutation analysis of APC and Asef demonstrated that APC-Asn507, APC-Asn550, Asef-Glu183, and Asef-Ala186 are critical in the interaction between APC and Asef. These works reveal the molecular mechanism by which APC recognizes Asef and provide a structural basis for the inhibitors design of APC-Asef interaction.

## Structure-Based Design of First-In-Class APC-Asef Inhibitors

**FIGURE 4:**
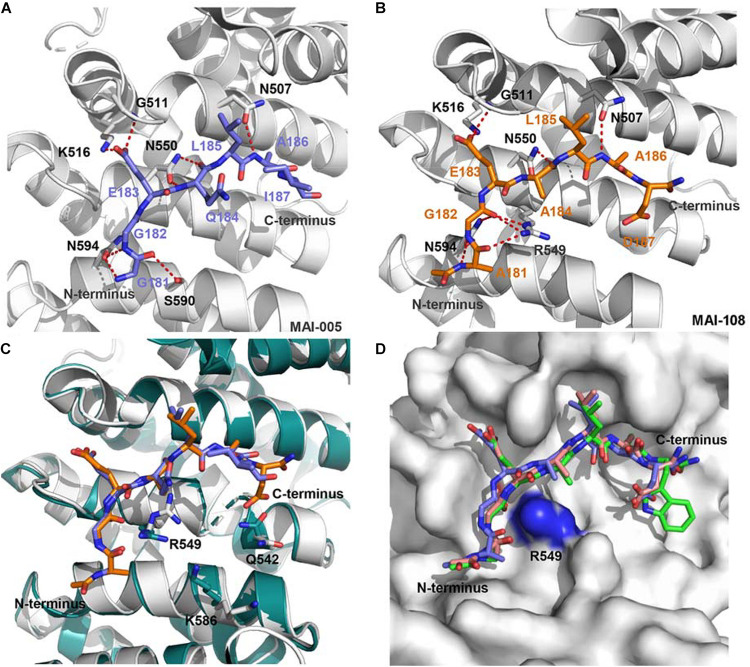
**(A)** The interactions of APC and MAI-005. APC is shown as a cartoon representation (gray), and MAI-005 is shown as a stick representation (purple). Red dashed lines represent hydrogen bonds. **(B)** Crystal structure of APC and MAI-108 (5IZ8). APC is shown as a cartoon representation (gray), and MAI-108 is shown as a stick representation (orange). Red dashed lines represent hydrogen bonds. **(C)** Structure comparison of APC/MAI-005 (APC, cartoon, gray; MAI-005, stick, purple) and APC/MAI-108 (APC, cartoon, cyan; MAI-108, stick, orange). **(D)** Superimposition of APC/MAI-102 (5IZA), APC/MAI-107 (5IZ9), and APC/MAI-108 (5IZ8) (APC, surface, gray; MAI-102, stick, green; MAI-107, stick, light pink; MAI-108, stick, purple).

Although the crystal structure of APC-Asef complex has been solved, the interaction area of APC-Asef is generally flat and large. It is still a challenge to develop inhibitors of APC-Asef interaction. To identify potent inhibitors of APC-Asef interaction, Jiang et al. (2017) first determined the hot spots of APC-Asef binding (Jiang et al., 2017). The structural analysis of the APC-Asef interaction revealed that the flexible segment of Asef-ABR (^176^SHPGGGGEQLAINELISDG^194^, referred to as ORIGIN) fit into the flat pocket of APC-ARM with 36 pairs of residue-residue interactions. Based on this ORIGIN sequence, Jiang et al. (2017) first designed a fluorescent probe (Ac-GGGGEQLAINELISDGK-FITC) and established a fluorescence polarization (FP) competitive assay. They synthesized various truncated peptides based on the ORIGIN, such as ^179^GGGGEQLAINELISDG^193^ (*K*_*i*_ = 17.1 ± 3.52 μM), ^181^GGEQLAINELISD^192^ (*K*_*i*_ = 49.1 ± 13.2 μM), ^81^GGEQLAI^187^ (*K*_*i*_ = 44.62 ± 0.99 μM), ^183^EQLAINEL^190^ (*K*_*i*_ > 300 μM), and ^187^INELISD^192^ (*K*_*i*_ > 300 μM). As a result, the peptide MAI-005 (^181^GGEQLAI^187^) with an acceptable activity (*K*_*i*_ = 44.62 ± 0.99 μM) and moderate size was considered as a hit peptide for further optimization (Figure 4A). They also replaced residues in each position of MAI-005 and synthesized these mutated peptides to establish a peptide library. High throughput screening of the peptide library by FP assay suggested that the favorable mutations at positions Gly181, Gln184, and Ile187 of MAI-005 could increase 4–14% binding affinities separately. Combining these favorable mutations, they developed three peptides MAI-102 (^181^GGEALAW^187^, *K*_*i*_ = 3.12 ± 0.70 μM), MAI-107 (^181^GGEALAD^187^, *K*_*i*_ = 3.80 ± 0.72 μM), and MAI-108 (^181^AGEALAD^187^, *K*_*i*_ = 2.41 ± 0.88 μM), with more than 12-fold increased affinity than MAI-005.

**FIGURE 5:**
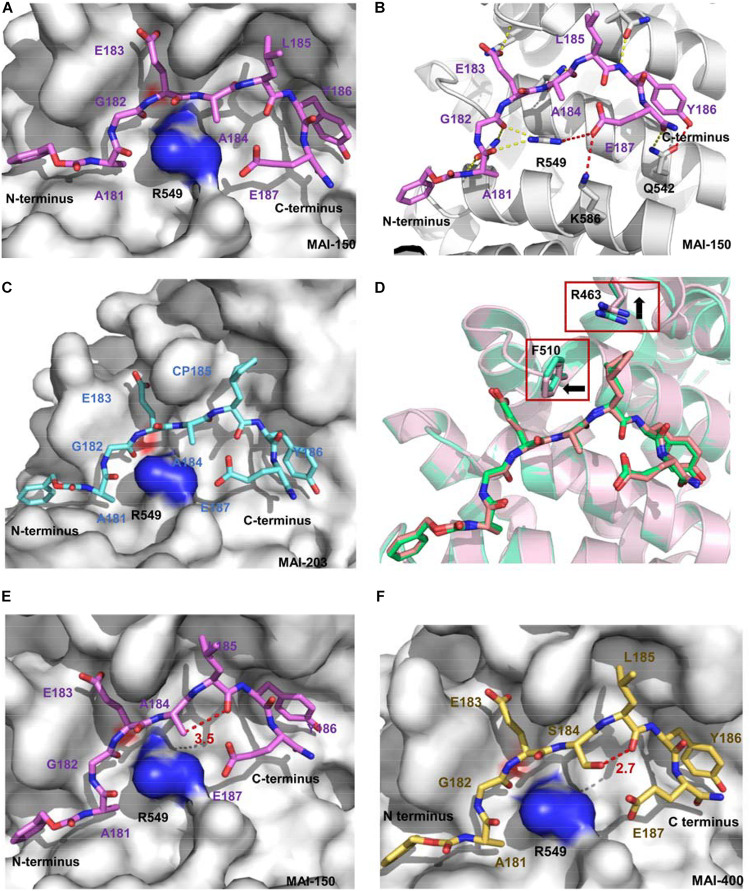
**(A)** Crystal structure of APC and MAI-150 (5IZ6). APC is shown as a surface representation (gray), and MAI-150 is shown as a stick representation (pink). **(B)** The interactions of APC and MAI-150 (APC, cartoon, gray; MAI-150, stick, pink). Yellow dashed lines represent hydrogen bonds. Red dashed lines represent new hydrogen bonds. **(C)** Crystal structure of APC and MAI-203 (5B6G). APC is shown as a surface representation (gray), and MAI-150 is shown as a stick representation (blue). **(D)** A structural comparison of APC/MAI-150 (APC, cartoon, pink; MAI-150, stick, pink) and APC/MAI-203 (APC, cartoon, cyan; MAI-203, stick, cyan). **(E)** Crystal structure of APC and MAI-150. APC is shown as a surface representation (gray), and MAI-150 is shown as a stick representation (pink). **(F)** Crystal structure of APC and MAI-400 (5Z8H). APC is shown as a surface representation (gray), and MAI-400 is shown as a stick representation (yellow). Red dashed lines represent hydrogen bonds.

The crystal structures of APC/MAI-102, APC/MAI-107, and APC/MAI-108 revealed that MAI-108 induced conformational changes in the APC-ARM pocket at the position of Arg549, Lys586, and Gln542. All three compounds could induce the conformational changes of the side chain of APC Arg549 (Figures 4B–D), resulting in a doughnut shape of the APC-ARM pocket with the more druggable property. Compared with the flat interaction surface of APC-Asef, the doughnut shape pocket of APC provided a potential druggable target for the development of APC-Asef inhibitors. Moreover, the overlapped structures of MAI-102, MAI-107, and MAI-108 in the binding site provided new information for the next optimization. The residues 181–185 of MAI-102, MAI-107, and MAI-108 had similar binding confirmation, but the C terminus exhibited diverse conformation (Figure 4D). The optimization of C-terminal peptide residues might improve the binding affinity via adding additional interactions. Therefore, they established a next-generation library of peptides based on the scaffold of ^181^AGEAL^185^. The new peptides library involved various N-terminal groups, entirely stochastic diverse residues at position 186, and the confined acidic or polar residues at position 187. Finally, they found MAI-150 (^181^AGEALYE^187^, *K_*i*_* = 0.12 ± 0.02 μM) was a better inhibitor of APC-Asef. The binding affinity of MAI-150 increased more than 20-fold in comparison to MAI-108 and 370-fold to MAI-005. The crystal structure and mutation analysis showed that the improved binding affinity might be caused by forming the additional interactions through residue Tyr186, Glu187, and the N-terminal benzyloxycarbonyl group of MAI-150 (Figures 5A,B).

Next, peptidomimetic inhibitors were designed to improve the binding affinity based on the APC/MAI-150 complex structure. The optimization of the N-terminal group and residues at positions 185 and 186 resulted in the peptidomimetic compound MAI-203 with cyclopentylalanine residue at position 185. The *K*_*i*_ value of MAI-203 to APC was as high as 0.015 ± 0.001 μM, equivalent to the dissociation constant of APC-Asef (*K*_*d*_ = 0.018 μM). The binding affinity of MAI-203 improved more than nine-fold compared to MAI-150 and 2,900-fold to MAI-005. The crystal structure of APC/MAI-203 was determined to explain the high binding affinity of MAI-203 to APC. MAI-203 induced the subsite formed by Arg463 and Phe510 of the APC larger to adapt to the cyclopentylalanine residue of MAI-203. This might facilitate tighter hydrophobic interactions and account for the improved binding affinity of MAI-203 (Figures 5C,D). The cell permeability of MAI-203 is low because of the large size and peptide properties. Jiang et al. (2017) connected the penetrating peptide TAT (trans-activating transcriptional activator) to the C-terminal of MAI-203 and MAI-150 and obtained the cell-permeable peptide MAIT-203 and MAIT-150.

To determine the effect of peptides *in vivo*, Jiang et al. (2017) performed co-immunoprecipitation experiments in HEK293T cells and SW480 cells. The results showed that MAI-203 and MAI-150 reduced the APC-Asef interaction in a dose-dependent manner. The cellular thermal shift assay in SW480 cells revealed that MAIT peptides could increase the thermal denaturation temperature (Δ*T_*m*_* = 6.92 ± 0.94°C), which indicated that the MAIT peptides directly interacted with APC in cells. Next, the function of MAIT peptides in CRC cells was investigated. RCTA, transwell and wound-healing assays were performed to evaluate the anti-migratory effect of MAIT peptides. The results showed that MAIT peptides at 10 μM caused significant repression of cell migration and invasion in SW480 and HCT116 cells. Cell vitality and proliferation experiments showed that MAIT peptides at concentrations up to 100 μM had no effect on the morphology or growth of SW480 and HCT116 cells. In the biology assays, MAIT-203 could effectively block the APC–Asef interaction, thereby inhibiting the migration and invasion of two types of CRC cells without affecting cell proliferation. In a fluorescence-based GEF assay with GTP-bound GTPases, MAIT-203 was used as a chemical probe to identify the downstream GTPase substrate of activated-Asef by APC-ARM. The results suggested that the GEF activity of CDC42 was stimulated by APC-Asef interaction and decreased by treatment of MAIT-203. Therefore, the downstream GTPase in the Asef activation pathway stimulated by APC was CDC42. Future research will focus on the mechanism of cell migration and invasion through the APC-Asef-CDC42 pathway.

## Optimization Strategy Leading to the Best-In-Class Inhibitor

To obtain highly effective new APC-Asef inhibitors, Yang et al. (2018) further optimized MAI-150 via a rational structure-based strategy. According to the complex structure of MAI-150/APC (PDB code 5IZ6), the hydrogen atom in the side chain of residue A184 and the oxygen atom in the backbone of residue L185 have a suitable angel (120.9°) and distance (3.5 Å) to form an intramolecular hydrogen bond (Figure 5E). Then, Yang et al. (2018) designed and synthesized a series of MAI-150 derivatives by replacing the A184 residue with polar amino acids, whose side chains provided hydrogen-bond donors. These efforts led to discovering MAI-400, a best-in-class APC-Asef inhibitor with a *K*_*d*_ of 0.012 μM and an IC_50_ of 0.25 ± 0.01 μM. The complex of APC/MAI-400 was determined, and the formation of intramolecular hydrogen bond was confirmed (Figure 5F). Surface Plasmon Resonance (SPR) and ITC assay revealed the binding affinity of MAI-400 to APC and explained the contribution of the intramolecular hydrogen bond. Co-immunoprecipitation (co-IP) assay showed that MAI-400 efficiently disrupted the APC-Asef interaction in a dose-dependent manner. Their successful strategy may be a useful approach for peptide optimization.

## Small Molecule Inhibitors of APC-Asef Protein-Protein Interaction

**FIGURE 6:**
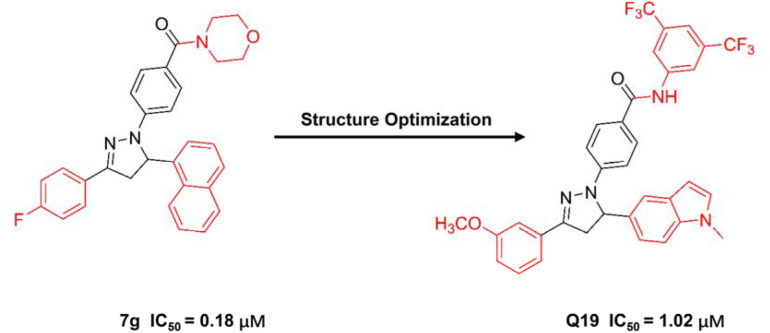
The structure of compound 7g and Q19.

Zhu et al. reported numbers of 2-H pyrazole derivatives containing morpholine groups as inhibitors of APC-Asef (Yan et al., 2019) (Figure 6). Based on the structure of APC-PreARM-ARM/Asef-ABR (PDB: 3NMZ), they found 2-H pyrazole derivatives showed good estimated binding free energy from −56.45 to −35.46 kcal/mol by molecular docking. Among these compounds, compound 7g had the best-estimated binding free energy of −56.45 kcal/mol. The estimated binding mode of 7g-APC suggested 7g bound to the central position of the APC-Asef interact surface through six conventional hydrogen bonds, two carbon-hydrogen bonds, and six Pi bonds. These predicted interactions reflected the stability of APC-7g binding.

Next, 2-H pyrazole derivatives 7a–7y were synthesized through a 5-step route. The structure of 7g was identified by NMR, MS, elemental analysis, and single-crystal X-ray diffraction analysis. The efficacies of derivatives 7a–7y were evaluated by the FP competitive assay. The IC_50_ values of these compounds ranged from 0.18 to 22.68 μM, and the typical compound 7g showed the best IC_50_ values of 0.18 μM. ITC furtherly verified the binding affinity of 7g-APC and 7g-Asef. The results showed that 7g could interact to APC with a *K*_*d*_ value of 12.8 μM

Compound 7g exhibited anti-proliferative activity against six cancer cell lines, especially against HCT-116 cells. Compound 7g also induced HCT-116 cell apoptosis at a concentration of 2–4 μM. Further studies showed that 7g could increase ROS level and induce apoptosis of HCT-116 cells. Compound 7g could weaken the adhesion and invasion of HCT-116 cells at a concentration of 2 μM. Besides the in vitro bioactivity, the in vivo anti-tumor efficacy of 7g was also investigated. The mouse xenograft and orthotopic transplantation models were established to evaluate the in vivo anti-tumor efficacy of 7g. The average weight of the excised tumor and tumor volume in 7g treated mice significantly reduced compared to the vehicle group. The results showed 7g significantly suppressed tumor growth.

Through further optimization of 7g, compound Q19 was obtained as the following generation inhibitor of APC-Asef with the IC_50_ values of 1 μM (Qi et al., 2020). Compound Q19 could inhibit the proliferation of HCT-116 cells with the GC_50_ values of 1.37 μM. Besides, compound Q19 could induce the mitochondria depolarization and apoptosis of HCT-116 cells. However, Q19 was not as potent as 7g in FP assay and anti-proliferation assay.

Based on these results, it is hard to determine the direct target of compounds 7g and Q19. The only evidence to verify the direct binding of APC and 7g is the ITC assays. However, the original data of ITC results suggest that the molar ratio is not less than 1, indicating that the APC is not saturated by 7g. It is theoretically impossible to calculate the accurate *K*_*d*_ value due to its atypical curve and appropriate molar ratio. The current *K*_*d*_ value of 7g to APC is not convincing, and the binding affinity of 7g-APC still needs further confirmation. More convincing binding affinity assays and co-crystal structure need to be provided to assure the target of 7g. Besides, the interaction of APC-Asef with ROS-induced apoptosis and mitochondria depolarization needs to be clarified.

Another group was interested in developing APC-Asef inhibitors and tried to identify new small molecular inhibitors of APC-Asef through a combination of docking methods and molecular dynamics simulations (Jadav et al., 2020). They analyzed and validated the central binding region of APC bind to MAI-peptide, including Arg539, Lys516, Asn507, and Trp553 of APC. Then, structure-based virtual screening (SBVS) of ZINC13, NCI, and Maybridge public compound library were carried out. The top-scoring hits were explored by induced fit docking analysis and molecular dynamic simulations. Finally, the 16 ligand hits with amide groups were predicted to be APC-Asef inhibitors with the pretty similarity of known anti-cancer β-carbolines and pharmacological profile. However, bioassays are still required to prove the actual targets and activities of these molecules.

## Bioassays for Evaluating the Binding Affinity and Efficacy of APC-Asef Inhibitors

### Binding Assays

Appropriate methods that can sensitively evaluate the binding affinity of screening candidates help identify and validate PPI inhibitors, including FP assay, ITC, SPR, Thermal shift assay (TSA), and cellular thermal shift assay (CETSA). In the initial screening process, an FP competitive assay was established to screen APC-Asef inhibitors. In this assay, the formation of the APC-inhibitor complex was deduced from a decrease in FP, and the IC_50_ and *K*_*i*_ values were determined. The FP assay is a convenient and effective competition binding assay for screening PPIs inhibitors. However, the applications of FP is limited to screening inhibitors that competitively bind to the receptors at a stoichiometry of 1:1 (Huang, 2003). As the gold standard technique for measuring direct binding affinity, ITC experiments are usually carried out in the following confirmation processes (Zeiss and Bauer-Brandl, 2006; Wiebke, 2015). ITC assays further confirmed the binding affinity of MAI-peptides to APC or Asef. The ITC results showed that the stoichiometry ratio of MAI-peptide and APC was 1:1, which was suitable for the FP assay. The *K*_*d*_ value measured by ITC is consistent with the IC_50_ and *K*_*i*_ value by FP. The enthalpy change and entropy change during the binding of peptides to APC provided useful information for hit-to-lead optimization. ITC is not suitable for high throughput screening. SPR is also used to determine the direct binding affinity and thermodynamic parameters of MAI-peptides to APC (Yan, 2015). The dissociation and association rate constants help to understand the structure-activity relationships (SAR) of MAI-peptides. TSA and CETSA are reliable methods to measure the interaction between MAI-peptides and APC by determining the melting temperature (*T*_*m*_) of the protein complex (Zhang and Monsma, 2010; Rogez-Florent et al., 2014; Letourneau et al., 2019; Henderson et al., 2020; Rodriguez-Furlan and Hicks, 2021). The results of TSA and CETSA showed that MAI-peptides directly bind to APC *in vitro*.

### Functional Assays

Co-immunoprecipitation (co-IP), Real-Time Cell Analysis (RCTA), wound-healing and Transwell assay were performed to determine the function of MAI-peptides in CRC cells. Co-IP experiments in HEK293T cells and SW480 cells demonstrated the inhibitory effect of MAIT-peptides on physiologically APC-Asef interaction. RCTA can detect electrical impedance through microelectronic biosensor technology and measure the migration and invasion of CRC cells. The RCTA results showed that MAIT-203 remarkably inhibited the migration and invasion of SW480 and HCT116 cells better than MAIT-150. The wound-healing assay was used to detect the influence of MAIT-peptides on the spreading tendency of SW480 and HCT116 cells. MAIT-203 significantly inhibited cell migration, especially at the leading edges. In addition, a Transwell assay was performed to confirm the anti-migration effect of MAIT-peptides. Consistent with the previous results, MAIT-203 reduced the migration of SW480 and HCT116 cells. To evaluate the anti-migration efficacy of MAIT-peptides *in vivo*, researchers need to establish a mouse model of CRC with liver metastasis. The following research may focus on the efficacy of APC-Asef inhibitors on CRC metastasis *in vivo*.

## Challenges in Targeting APC-Asef PPI

Targeting the APC-Asef interaction using peptidomimetic inhibitors as a new therapy for CRC migration has achieved exciting results. MAIT-203 and its derivatives have been found to inhibit the migration and invasion of SW480 cells efficiently. However, undesirable physicochemical properties of peptides, such as poor oral bioavailability, low permeability, and instability, limit their further development.

The Asef binding site on APC is tremendous, and it is still a challenge to block this site with small molecules. Small molecule inhibitors possess several distinct advantages, such as higher membrane permeability, better selectivity, and higher affinity than peptides. With the current complex structures of peptide-APC, the discovery of small-molecule inhibitors of APC-Asef might be feasible. The hot spots in APC and the binding mode of peptides provide essential information for the design of small-molecule inhibitors. Peptides simplification also provides an available method for the design of small molecule inhibitors.

Besides, allosteric modulation could provide a new strategy to regulate PPI via conformational changes to avoid the flat and large interaction surface of PPI. Allosteric inhibitors targeting APC might be another way to discover small molecule inhibitors. Most allosteric PPI modulators were discovered fortuitously. However, the rapid progress in chemical and structural biology facilitates the rational design of allosteric PPI modulators. The first step is to identify the allosteric site of APC-Asef based on the APC-Asef co-complex structure. Multiple computational approaches are feasible to predict allosteric sites, such as statistical coupling analysis (SCA) (Lockless and Ranganathan, 1999), FRpred (Fischer et al., 2008), ConSeq (Berezin et al., 2004), ConCavity (Capra et al., 2009), and Allosite (Huang et al., 2013). These methods help us analyze and select potential allosteric sites of APC-Asef. Once the allosteric site is uncovered, virtual screening of allosteric modulators and standard-precision (SP) docking are performed. The commercial screening hits are chosen based on the comprehensive analysis of their binding modes and physiochemical properties. Finally, experimental screening and site validation could yield the possible hit compounds as the allosteric inhibitors of APC-Asef. These rational design strategies might inspire other research of allosteric PPI-based drug discovery.

Degradation of truncated APC by PROTACs technology might disturb the APC-Asef interaction and inhibit CRC migration, which provides a new method for CRC therapy.

Additionally, the APC-Asef interaction is regulated by HGF and phosphatidylinositol-3-kinase. Multiple-target therapy might be a more efficient method in cancer treatment. Therefore, the combination of APC-Asef interaction inhibitors and HGF inhibitors, as well as PI3K inhibitors, might be tolerable and effective for the treatment of CRC.

The APC-Asef interaction could be a promising target for CRC therapy. The development of APC-Asef interaction inhibitors is still in the early stages. The discovery of druggable APC-Asef inhibitors is needed for CRC therapy. Further studies will focus on the preclinical studies of APC-Asef inhibitors to provide new options for the treatment of CRC.

## Conclusion

Protein-protein interactions are attractive drug targets in many diseases. Most of the disease-relevant PPIs are considered undruggable because of their flat, featureless and expansive interface. The structure-based design has been an important strategy for discovering PPI inhibitors. The discovery of APC-Asef inhibitors is an example of rational design utilizing PPI structural information. Firstly, the crystal structures of APC-Asef provided a molecular basis for rational inhibitor discovery. Hotspot identification helped to characterize the key binding site and served as the starting point for the optimization of APC-Asef inhibitors. Next, mutagenesis and structural studies of APC-lead peptide provided the SAR. Multiple optimizations based on the structure resulted in the desirable inhibitor with high binding affinity. Finally, the evaluation of the binding affinity and efficacy of APC-Asef inhibitors *in vitro* and *in vivo* not only demonstrated that APC-Asef PPI was a druggable target of metastatic CRC but also provided a potential lead inhibitor for CRC drug discovery. The successful discovery of APC-Asef inhibitors validated an effective paradigm in structure-based PPI inhibitors design. It provided proof-of-principle for other PPI programs and enabled the development of numerous drugs that target PPIs.

## Author Contributions

SL, XY, and JZha conceived the study. XY reviewed and edited the manuscript. JZho, QZ, and LF contributed analysis tools. All authors wrote the manuscript.

## Conflict of Interest

The authors declare that the research was conducted in the absence of any commercial or financial relationships that could be construed as a potential conflict of interest.
